# Supplementary material for: Pseudocell Tracer—A method for inferring dynamic trajectories using scRNAseq and its application to B cells undergoing immunoglobulin class switch recombination
Source: PLoS Comput Biol. 2021 May 3;17(5):e1008094. doi: 10.1371/journal.pcbi.1008094 (PMC8118552; doi:10.1371/journal.pcbi.1008094)
Supplement: S1 Table — (DOCX) [file pcbi.1008094.s006.docx]

**Supplementary Table 1. Sample size analysis**

| **Sample_Size=** | **500** | **1000** | **2500** | **5000** |
| --- | --- | --- | --- | --- |
| Ighm | 0.207 | 0.326 | 0.414 | 0.464 |
| Ighg1 | 0.168 | 0.282 | 0.326 | 0.335 |
| Ighg2b | 0.174 | 0.138 | 0.21 | 0.286 |
| Ighg3 | 0.042 | 0.137 | 0.156 | 0.159 |
| Aicda | 0.626 | 0.632 | 0.678 | 0.713 |
| Ung | 0.427 | 0.431 | 0.539 | 0.566 |
| Bcl6 | 0.071 | 0.239 | 0.218 | 0.216 |
| Foxo1 | 0.008 | 0.067 | 0.11 | 0.148 |

*(Pearson correlations)
