# Supplementary material for: Pseudocell Tracer—A method for inferring dynamic trajectories using scRNAseq and its application to B cells undergoing immunoglobulin class switch recombination
Source: PLoS Comput Biol. 2021 May 3;17(5):e1008094. doi: 10.1371/journal.pcbi.1008094 (PMC8118552; doi:10.1371/journal.pcbi.1008094)
Supplement: S2 Table — (DOCX) [file pcbi.1008094.s007.docx]

**Supplementary Table 2. Hyperparameter table**

| HyperParameters |  |
| --- | --- |
| Encoder Hidden Layer 1 | 1024 |
| Encoder Hidden Layer 2 | 512 |
| Latent Layer | 256 |
| Decoder Hidden Layer 1 | 512 |
| Decoder Hidden Layer 2 | 1024 |
| Translation Layer (Side information) | 512 |
| L2 Norm | 0.0001 |
| Learning Rate | 0.0001 |
|  |  |
| CGAN Z Dim (Noise) | 32 |
| Generator Layer 1 | 512 |
| Generator Layer 2 | 512 |
| Discriminator Layer | 512 |
| Learning Rate | 0.00005 |
| Epochs | 5000 |
